# Concomitant Systemic Lupus Erythematosus and Glanzmann Thrombocytopenia: A Case Report and Literature Review

**DOI:** 10.1155/crrh/1543762

**Published:** 2025-04-15

**Authors:** Maryam Noory, Haniyeh Hajian, Farzad Kompani, Azadeh Kiumarsi, Mohammad Shahrooei, Vahid Ziaee

**Affiliations:** ^1^Children's Medical Center, Pediatrics Center of Excellence, Tehran, Iran; ^2^Department of Pediatrics, Tehran University of Medical Science, Tehran, Iran; ^3^Clinical and Diagnostic Immunology, Department of Microbiology, Immunology, and Transplantation, KU Leuven, Leuven, Belgium; ^4^Pediatric Rheumatology Society of Iran, Tehran, Iran; ^5^Pediatric Rheumatology Research Group, Rheumatology Research Center, Tehran University of Medical Science, Tehran, Iran

**Keywords:** children, glanzmann thrombasthenia, immune-mediated platelet disorders, systemic lupus erythematosus

## Abstract

**Background:** Glanzmann thrombasthenia (GT) is a rare disease that manifests with bleeding in different parts such as epistaxis and bruising. GT can be congenital or acquired. Systemic lupus erythematosus (SLE) is an autoimmune disorder. It is mentioned that the acquired type can be associated with other disorders like malignancies and autoimmune disorders. There is no report about the co-occurrence of congenital GT with SLE.

**Case Report:** In this report, we present this co-occurrence in a girl. An 11-year-old girl was referred to our clinic with severe and uncontrolled epistaxis. She had a history of recurrent epistaxis, gastrointestinal bleeding, and bruising. She also had a malar rash and generalized body pain. She was admitted, and after clinical and laboratory assessments, a co-occurrence of congenital GT with SLE was diagnosed.

**Conclusion:** The co-occurrence of congenital GT and SLE has not been reported until now. Patients with this presentation should be closely followed up because the risk of bleeding is high for them.

## 1. Introduction

Glanzmann thrombasthenia (GT) is a rare congenital disorder with an incidence rate of about 1 per million in the general population. The main pathogenesis of the disease is the loss of integrin alpha IIb beta 3 on the platelet, which is a receptor for platelets that facilitates platelet aggregation and hemostasis [1–3]. The primary clinical manifestation of GT is recurrent bleeding, particularly in the mucocutaneous membrane [3, 4].

Clinical presentation may vary from mild to asymptomatic, with epistaxis being the most prevalent clinical manifestation in children [5]. Other common manifestations include menorrhagia, gingival bleeding, and gastrointestinal bleeding. The mean age of clinical presentation is reported to be 5.6 years, with most cases presenting in 14-year-olds [3].

GT has two types: congenital and acquired. The acquired type may be associated with autoimmune disorders, but there is limited data on the co-occurrence of congenital GT with other disorders such as autoimmune disorders [6, 7].

Systemic lupus erythematosus (SLE) is an autoimmune disorder with a female predominance that affects the innate and adaptive immune systems. The exact etiology of SLE is not fully understood [8, 9], but it is known that thrombocytopenia in SLE can range from mild to severe and may be associated with autoantibodies against platelets, antiphospholipid antibody syndrome, and glycoprotein IIb/IIIa. Further studies are needed to explore associations of SLE with other disorders [10, 11].

In this report, we present the case of an 11-year-old girl with both GT and SLE.

## 2. Case Presentation

An 11-year-old girl was referred to our clinic with severe and uncontrolled epistaxis that had been ongoing for 2 h. The patient had a history of recurrent bruising without any known trauma or identifiable cause since the age of 3 months. She was diagnosed with GT and hemophilia type 1 at the age of 3. Over the years, she had multiple hospitalizations due to episodes of epistaxis and gastrointestinal bleeding, for which she received packed cell and factor VII infusions to manage the bleeding.

The patient, the first child of consanguineous parents, had a family history of occasional epistaxis in her father and uncles. Upon arrival at our center, she was hospitalized due to the severe and uncontrolled epistaxis, which was successfully managed with nasal tranexamic acid.

In addition to bruising and bleeding, the patient presented with generalized body pain and a malar rash before being referred to our center. She was initially seen by a physician who then referred her to our clinic for further evaluation for SLE.

During the physical examination, a malar rash was observed on her cheeks, along with pale sclera. No other significant signs or symptoms were noted in the musculoskeletal system examination.

Blood tests were conducted to assess the coagulation system and screen for SLE. The results are detailed in Table 1, confirming the diagnosis of SLE based on laboratory findings. Other lab tests including liver function tests, BUN and creatinine, PT, PTT, lupus anticoagulant, antiphospholipid antibodies, coombs (direct, indirect) and were normal. Flow cytometry also confirmed congenital GT.

Genetic testing revealed a mutation in ITGB3 (NM_000212:c.125 + 30C > T, rs751462011, AD, 619271). Treatment was started with steroid pulse therapy (30 mg/kg/day for 3 days) and then continued with maintenance therapy (1 mg/kg/day) and hydroxychloroquine (5 mg/kg/day). Fresh frozen plasma (FFP) was infused for the patient (10 cc/kg) once. The patient was discharged in stable condition with outpatient follow-ups recommended. Calcium (500 mg/daily) and vitamin D supplements (50000 IU monthly) were added to the treatment as complementary therapy for the prevention of osteopenia. Moreover, factor VII infusion was recommended if bleeding occurred. The treatment details are shown in Table 2.

After 2 months, the patient showed improvement in clinical symptoms (skin rash and musculoskeletal pain) and the dosage of prednisolone was gradually decreased to 0.75 mg/kg/day, and after 4 months to 0.5 mg/kg/day. Other treatments continued without any changes. All disease activity parameters in the laboratory (WBC, ESR, platelet count, anti ds-DNA, and complements) were normal after 4–12 months of treatment (Table 1).

Over a year of follow-up, the bleeding episodes decreased and factor VII infusion was needed only once during the first 6 months after starting the treatment of SLE, and in the second 6 months she did not need factor VII infusion. No important adverse effects were reported by the parents during the 1-year follow-up, except for an increase of about 3 kg in body weight in the 4 months after steroid therapy.

## 3. Discussion

In this report, we present a case of congenital GT that was confirmed with a genetic test showing a mutation in the ITGB3 gene and was associated with SLE. There is one report of acquired GT accompanying SLE [12]. To our knowledge, there is no report of congenital GT co-occurring with SLE, making this the first such report worldwide.

Congenital GT is a rare disease with an autosomal recessive genetic pattern. Mutations in genes such as ITGA2B and ITGB3 can cause congenital GT [13]. Our case involved congenital GT with a positive genetic test for ITGB3. Additionally, a positive familial history was noted in our case, with a history of epistaxis.

Alpha IIb/beta3 integrin, a glycoprotein affected in this disease, leads to coagulopathy. Patients with GT experience recurrent bleeding in various sites without apparent causes, such as epistaxis and bruising [13, 14]. Our patient had a history of recurrent epistaxis, gastrointestinal bleeding, bruising, and hematuria. The hematuria in our patient was attributed to GT since SLE-related hematuria is typically accompanied by proteinuria, which was not present in our case. SLE was diagnosed based on malar rash, decreased serum complements (C3 and C4), positive antineuclear antibodies (ANA), positive anti ds-DNA, thrombocytopenia, and hematuria [15].

Patients with GT have a normal platelet count but nonfunctional platelets [6]. Thrombocytopenia in our case may have been induced by SLE, as SLE can cause thrombocytopenia but not GT [16]. The co-occurrence of SLE and GT resulted in thrombasthenia and thrombocytopenia in our patient. Our patient had low nonfunctional platelets. Corticosteroids and hydroxychloroquine were prescribed to induce remission in SLE and increase the platelet count.

Flow cytometry is a useful method for diagnosing GT, typically showing defects in alpha IIb/beta3 integrin including CD41 (alpha IIb) and CD61 (beta3) [17]. Flow cytometry in our case revealed low levels of CD41 (3%) and CD61 (0.1%), consistent with GT.

Treatment is based on the severity of bleeding. Local measures like pressure application, sutures, cauterization, and ice packs may suffice for mild bleeding. For more severe cases, tranexamic acid and factor VII can be used [4, 5, 18]. In our case, these options were administered based on the severity of bleeding. For patients with recurrent severe bleedings and poor quality of life, hematopoietic stem cell transplant is a curative treatment option [5].

Although lupus in children can have a genetic basis, a mutation in ITGB3 has not been reported to produce monogenetic lupus. Therefore, there appears to be no direct connection between SLE and congenital GT. On the other hand, GT can develop as an autoimmune disorder as an acquired disease. In acquired GT, autoantibodies are produced by the immune system against the IIb/IIIa receptors that normally bind to fibrinogen [19]. Acquired GT can occur in association with SLE or Evans syndrome and antiphospholipid syndrome [12, 20], but this hypothesis was not considered for our patient because she has had episodes of bleeding since she was 1 year old and genetic positive study for the GT mutation.

However, our experience suggests that in cases of simultaneous GT and SLE, controlling lupus can effectively reduce and manage bleeding episodes.

## 4. Conclusion

The co-occurrence of congenital GT and SLE has not been reported until now. To the best of our knowledge, this is the first report of this presentation. Physicians should be aware of the association between congenital GT in children with SLE with normal platelet counts and severe bleeding. Good control of SLE may reduce bleeding episodes in the co-occurrence of congenital GT and SLE.

## Figures and Tables

**Table 1:** Laboratory data of the patient.

Test		Result (trends)	After 4 months	After 12 months
Complete blood count (CBC)	White blood cells (Nl > 4000)	1980	11,500	7000
	Red blood cells (Nl > 4,500,000)	2,300,000	3,900,000	4,540,000
	Hemoglobin (Nl > 12)	8.3	10.9	12
	Platelets (Nl > 150,000)	96,000	387,000	448,000

Inflammatory biomarkers	Erythrocyte sedimentation rate (Nl < 15)	108	25	11
	C-reactive protein (Nl < 9)	2	5	3.2

Coagulation tests	Bleeding time (BT)	> 15	—	—
	Factor XIII	Normal	—	—
	VwF	101%	—	—
	Prothrombin time (INR) (Nl: 13.5; 1)	13.6 (1.01)	13 (1)	—
	Partial thromboplastin time (Nl < 34)	30	34	—

Antinuclear antibody (ANA) (Nl < 1:80)		1:1280 (homogenous)		—

Anti-ds-DNA (Nl < 100)		1388	210	67

Complements	C3 (Nl > 80)	28	134	131
	C4 (Nl > 13)	3	26	17
	CH50 (Nl > 90)	Undetectable	125	110

Extractable ANA	Nucleosomes	Positive	—	—
	Histones	Positive	—	—
	Ribosomal-P	Positive	—	—
	AMA-M2	Borderline	—	—

Flow cytometry	CD41	3%	—	—
	CD42b	0.1%	—	—
	CD61	0.1%	—	—

Urine analysis	Hematuria	2+	Neg	Neg
	Protein	Neg	Neg	Neg
	Protein/creatinine	0.6	0.2	0.2

**Table 2:** Treatment protocol based on start and maintenance drugs.

Treatment	Start	Continue	Now
Fresh frozen plasma (FFP)	2 times (10 cc/kg) stat (2 days in a raw)	—	—
Steroid pulse therapy (methylprednisolone)	30 mg/kg/day (for 3 days in a row)	—	—
Prednisolone	1 mg/kg/day	0.75 and 0.5 mg/kg/day	0.25 mg/day
Hydroxychloroquine	6 mg/kg/day	6 mg/kg/day	6 mg/kg/day
Vitamin D 3	50,000 unit monthly	50,000 unit monthly	50,000 unit monthly

## Data Availability

The data used to support the findings of this study are available from the corresponding author upon request.
